# First record of *Rhodnius nasutus* Stål, 1859 (Hemiptera: Reduviidae: Triatominae) in an urban residential environment in the municipality of Barra, Bahia

**DOI:** 10.1590/S1984-29612026005

**Published:** 2026-04-20

**Authors:** Mauricio dos Santos Conceição, Milena Oliveira Albuquerque, Milena de Oliveira Almeida, Alice dos Santos Saraiva, Alini Dias de Pauda, Ricardo Lustosa Brito, Jairo Torres Magalhães-Junior, Flavia dos Santos

**Affiliations:** 1 Universidade Federal do Oeste da Bahia – UFOB, Centro Multidisciplinar de Barra – CMB, Laboratório de Saúde Única – LASU, Barra, BA, Brasil; 2 Universidade Federal do Oeste da Bahia – UFOB, Centro Multidisciplinar de Barra – CMB, Barra, BA, Brasil

**Keywords:** Chagas disease, entomological surveillance, Trypanosoma cruzi, Triatomines, Doença de Chagas, vigilância entomológica, Trypanosoma cruzi, Triatomíneos

## Abstract

Triatomines of the genus *Rhodnius* are of high epidemiological relevance in Brazil, being predominantly found in wild environments, especially in palm trees and nests. The aim of this study was to report, for the first time, the occurrence of *Rhodnius nasutus* in the domestic environment of the urban area of the municipality of Barra, Bahia. *R. nasutus* was collected inside an urban residence at different times, one female and one male. Coprological analysis indicated the presence of flagellated forms suggestive of *Trypanosoma cruzi* only in the female specimen. The presence of *R. nasutus*, a species typically associated with wild ecotopes of the Caatinga biome, reinforces its ability to disperse to transition areas between Caatinga and Cerrado, such as the municipality of Barra. This unprecedented record highlights the need for further investigation into food sources, dispersal capacity, and the species' potential for adaptation to the domestic environment.

## Resumo

Triatomíneos do gênero *Rhodnius* possuem elevada relevância epidemiológica no Brasil, sendo registrados predominantemente em ambientes silvestres, especialmente em palmeiras e ninhos. O objetivo deste estudo foi relatar, pela primeira vez, a ocorrência de *Rhodnius nasutus* no ambiente domiciliar da zona urbana do município de Barra, Bahia. Uma fêmea e um macho de *R. nasutus* foram coletados no intradomicílio de uma residência urbana em momentos distintos. A análise coprológica indicou a presença de formas flageladas sugestivas de *Trypanosoma cruzi* apenas no exemplar fêmea. A presença de *R. nasutus*, espécie tipicamente associada a ecótopos silvestres do bioma Caatinga, reforça sua capacidade de dispersão para áreas de transição entre Caatinga e Cerrado, como o município de Barra. Este registro inédito evidencia a necessidade de investigações adicionais sobre fontes alimentares, capacidade de dispersão e potencial de adaptação da espécie ao ambiente domiciliar.

## Introduction

Chagas disease (CD) is a parasitic anthropozoonosis caused by the protozoan *Trypanosoma cruzi* (Chagas, 1909). It is considered a neglected disease, as it mainly affects marginalized populations with high socioeconomic vulnerability. It occurs throughout Latin America, with Brazil standing out as one of the countries with the highest prevalence of the disease (Dias et al., 2016). Vector transmission of CD occurs through the passage of the protozoan from triatomine excreta through broken skin or mucous membranes of humans during or immediately after the triatomines' blood meal. There are also other forms of transmission: through the ingestion of food contaminated with parasites; mother-to-child or congenital transmission; through blood transfusions and organ transplantation; and through laboratory accidents (Dias et al., 2016). Currently, there are 159 different known species of triatomines worldwide, distributed into 19 genera and five tribes (Barbosa-Silva et al., 2026; Paiva et al., 2025). In Brazil, there are 65 species of triatomines, and in Bahia, 26 species have been recorded (Sousa et al., 2020); these species are associated with sylvatic, intradomiciliary, and peridomiciliary environments. The genera considered of greatest importance from an epidemiological point of view are *Panstrongylus*, *Rhodnius*, and *Triatoma*.

The *Rhodnius* genus comprises 20 species: *Rhodnius amazonicus* Almeida, Santos, Sposina, 1973; *Rhodnius barretti* Abad-Franch, Pavan, Jaramillo-O, Palomeque, Dale, Chaverra, Monteiro, 2013; *Rhodnius brethesi* Matta, 1919; *Rhodnius colombiensis* Mejia, Galvão, Jurberg, 1999; *Rhodnius dalessandroi* Carcavallo, Barreto, 1976; *Rhodnius domesticus* Neiva, Pinto, 1923; *Rhodnius ecuadoriensis* Lent , León, 1958; *Rhodnius marabaensis* Souza, Atzingen, Furtado, Oliveira, Nascimento, Vendrami, Gardim , Rosa, 2016; *Rhodnius micki* Zhao, Galvão , Cai, 2021; *Rhodnius milesi* Carcavallo, Rocha, Galvão , Jurberg, 2001; *Rhodnius montenegrensis* Rosa, Rocha, Gardim, Pinto, Mendonça, Ferreira Filho, Carvalho, Camargo, Oliveira, Nascimento, Cilense, Almeida, 2012; *Rhodnius nasutus* Stål, 1859; *Rhodnius neglectus* Lent, 1954; *Rhodnius neivai* Lent, 1953; *Rhodnius pallescens* Barber, 1932; *Rhodnius paraensis* Sherlock, Guitton, Miles, 1977; *Rhodnius pictipes* Stål, 1872; *Rhodnius prolixus* Stål, 1859; *Rhodnius robustus* Larrousse, 1927; *Rhodnius stali* Lent, Jurberg, Galvão, 1993. In the study by Oliveira-Correia et al. (2024), the species *Rhodnius zeledoni* Jurberg, Rocha, Galvão, 2009 underwent a taxonomic reevaluation and was considered as a synonym of *R. domesticus*. Species of the *Rhodnius* genus can reach a total length between 11 and 26 mm and possess chromatic variation ranging from yellowish-brown to black with dark brown or brownish-black spots (Lent & Wygodzinsky, 1979), this variation is associated with palm tree-inhabiting species (Dias et al., 2011).

Records of *R. nasutus* in its natural habitat match areas characterized by vegetation typical of the Caatinga biome (cacti, low and twisted thorny shrubs, dry appearance, and small leaves); there are also records in the Cerrado/Caatinga transition region with open arboreal vegetation, presenting elements typical of the Cerrado, Amazon, and Atlantic Forest (Dias et al., 2008). In sylvatic ecotopes, *R. nasutus* is found naturally inhabiting bird and mammal nests and *Copernicia prunifera* Moore, 1963 (carnaúba) palm trees, which can be located in the vicinity of human dwellings (Lima & Sarquis, 2008). In addition to these locations, *R. nasutus* can also occur in *Attalea speciosa* Martius, 1826 (babaçu), *Mauritia flexuosa* Younger, 1782 (buriti), *Syagrus oleracea* Beccari, 1916 (guariroba), and *Acrocomia intumescens* Drude, 1881 (macaúba-barriguda) palms (Dias et al., 2008; Abad-Franch et al., 2015), as well as in *Licania rigida* Martius, 1843 (Oiticica) dicotyledonous trees, which are typical of northeastern Brazil (Lima & Sarquis, 2008). Artificial ecotopes reported with the presence of adult *R. nasutus* insects were chicken coops, corrals, perches, pigsties, and piles of bricks, tiles, and wood (Sarquis et al., 2012). Given the above, the objective of this study is to report the first occurrence of *R. nasutus* in a household in the urban area of the municipality of Barra-BA, including a specimen infected with *Trypanosoma* sp.

## Material and Methods

**Figure 1:**
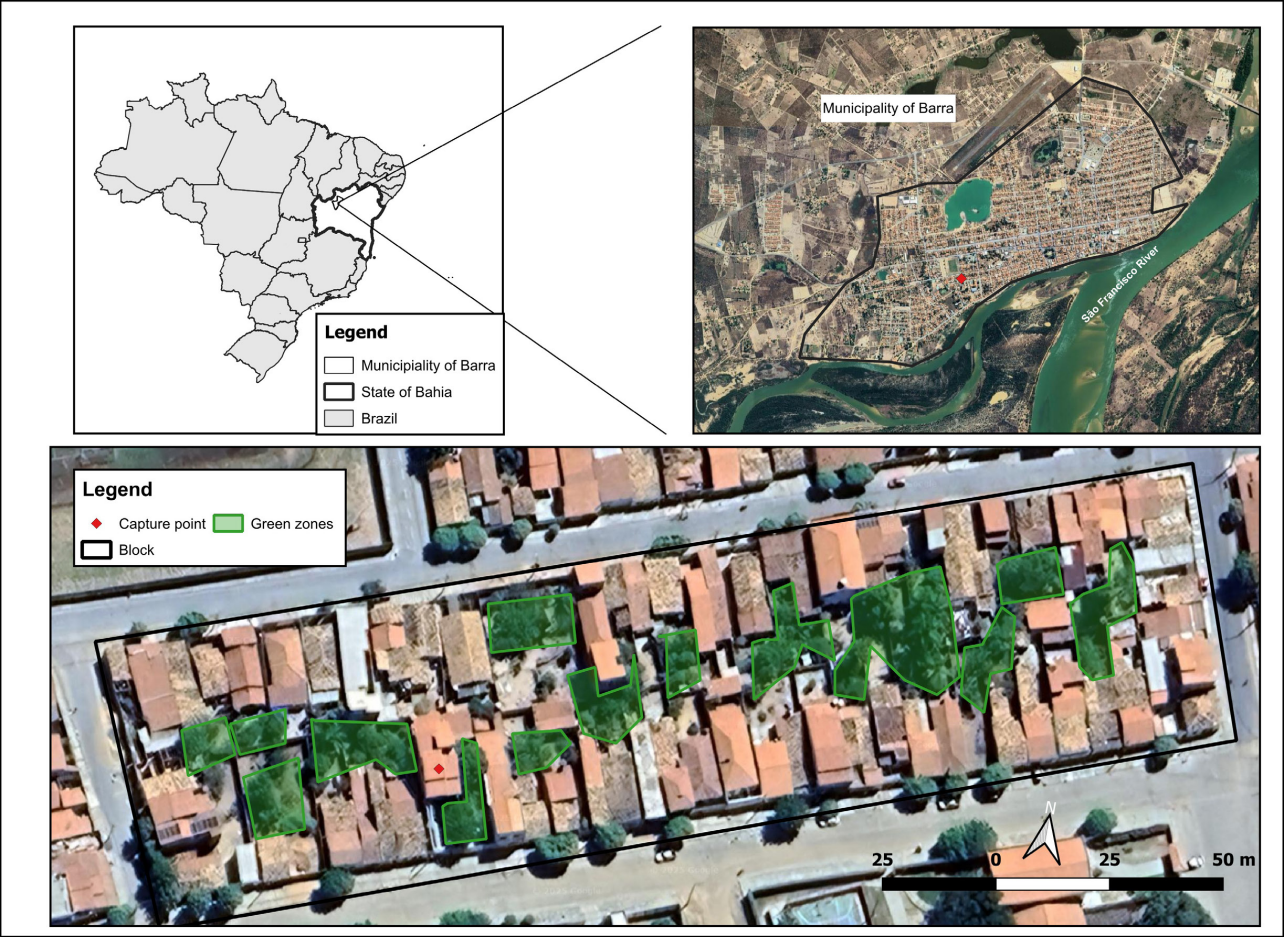
Geographic location of Brazil with emphasis on the State of Bahia, highlighting the municipality of Barra and the city block where the specimens were collected, showing the proximity of the urban residence to local green areas.

This qualitative and descriptive study was carried out in a household in the urban area of Barra (Figure 1), Bahia, Brazil, a municipality located in the Caatinga biome with a semi-arid climate. The area map was prepared in QGIS 3.44 using IBGE cartographic bases. Two triatomine bugs were passively collected by a resident of the same household on November 6th and December 9th, 2023, and sent to the local Epidemiological Surveillance. The first triatomine bug was collected from the living room floor in the morning, while the second triatomine bug was found flying in the kitchen, also in the morning. The household was located on the second floor of a building and was physically well-structured, with no cracks in its walls, lined, and with glass windows that were kept closed at night to prevent insects from entering and the family's only cat from escaping. There was no accumulated material inside the house because to prevent cockroach infestation, and it was cleaned twice a week. The peridomiciliary consisted only of a covered service area enclosed by a window and a small balcony with a few ornamental plants. The lights in the peridomestic areas were rarely turned on. This dwelling was located about 1km from the Rio Grande, a tributary of the São Francisco River, and surrounded by fruit trees from the peridomiciliary environments of neighboring houses.

Upon the first notification, an active intradomiciliary and peridomiciliary search was performed by an endemic disease control agent. The active search for triatomine bugs was carried out during the morning, using gloves, tweezers, a flashlight, and a suitable container to collect the insects. All rooms of the house were inspected in a clockwise direction (left to right), checking under mattresses, behind furniture, and in crevices that could harbor the vectors; upon the second one, the active search was expanded to all houses in the city block, thus increasing the possibility of capturing the triatomine bugs (Santos et al., 2022).

Taxonomic identification was based on morphological characteristics, according to Lent & Wygodzinsky (1979), with the aid of a stereomicroscope. The coproparasitological examination was conducted using fresh wet mounts from feces obtained by abdominal compression, homogenized in saline solution (0.9%), and observed in duplicate under optical microscopy (400x). The slides were subsequently stained with Panoptic to identify the presence of *Trypanosoma* sp*.* No molecular biology or isolation techniques (such as blood culture) were performed to identify the parasite species, since triatomines can be infected by *T. cruzi*, *Trypanosoma rangeli*, and other trypanosomatids (Ribeiro et al., 2019).

## Results

**Figure 2:**
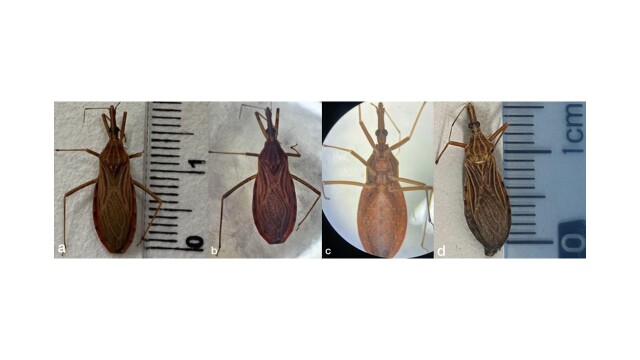
(A) *Rhodnius nasutus* female specimen measuring 17 millimeters; (B) Dorsal view of the female; (C) Ventral view of the female; (D) *Rhodnius nasutus* male specimen measuring 16 millimeters.

**Figure 3:**
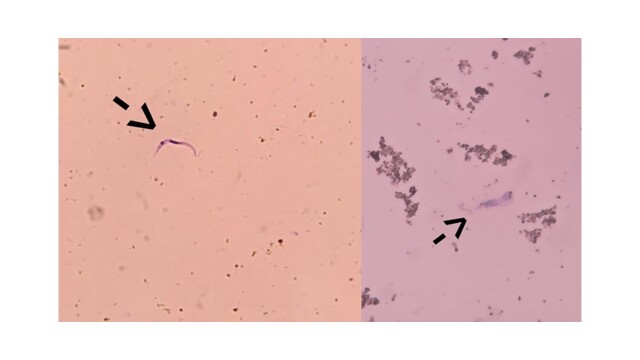
Flagellated forms suggestive of *Trypanosoma cruzi* detected in the feces of a female *Rhodnius nasutus* specimen, which was collected in the intradomicile of a house in the urban area of the municipality of Barra, Western Bahia, in November 2023.

Following the active search conducted by endemic disease control agents in the focal household, as well as in the 35 households within the city block, no triatomines were captured other than those collected by the resident. The taxonomic identification key confirmed the specimens as two *R. nasutus*: a female measuring 17 millimeters and a male measuring 16 millimeters (Figure 2). Of the two specimens submitted to coproparasitological examination, one female was engorged (with food in her gastrointestinal tract), while the male had no food present in his abdomen. Furthermore, the resident reported that she only saw the triatomine bug in her kitchen because it was flying around a lot. Only the female presented flagellated forms suggestive of *T. cruzi* in her feces (Figure 3).

## Discussion

This study reports the first occurrence of *R. nasutus* in a household in the urban area of the municipality of Barra-BA, including a specimen possibly infected with *T. cruzi. Rhodnius nasutus* is characterized as one of the main triatomine species associated with the Caatinga biome, with wide distribution in the Brazilian Northeast and importance in the *T. cruzi* transmission chain. Studies report its distribution in the states of Ceará, Bahia, Piauí, Rio Grande do Norte, Minas Gerais, Maranhão, Paraíba, and Pernambuco (Dias et al., 2008; Gurgel-Gonçalves et al., 2012). In Bahia, this species has already been described in the municipalities of Feira de Santana, São Domingos, and Curaçá (Gurgel-Gonçalves et al., 2012). In this context, the present research is proven significant, considering that other studies previously conducted in the municipality did not report the occurrence of this species (Santos et al., 2022).

The municipality of Barra presents environmental characteristics that favor the occurrence and dispersal of triatomines, as it is situated in a transition zone between the Cerrado and Caatinga biomes, in addition to possessing water barriers such as the São Francisco and Rio Grande rivers, streams, and circulation routes that may facilitate insect displacement between different areas (Gurgel-Gonçalves et al., 2012). In the period from 2009 to 2019, 12 different triatomine species were captured in the municipality of Barra, namely, *Triatoma sordida* Stål, 1859; *Triatoma pseudomaculata* Corrêa, Espínola, 1964; *Triatoma guazu* Lent, Wygodzinsky, 1979; *Triatoma infestans* Klug, 1834; *Triatoma pessoai* Galvão, Lent, 1959; *Triatoma brasiliense* Neiva, 1911; *R. neglectus*; *Panstrongylus diasi* Pinto, Lent, 1946; *Panstrongylus lutzi* Neiva, Pinto, 1923; *Panstrongylus megistus* Burmeister, 1835; *Panstrongylus lingnarius* Walker, 1873; and *Eratyrus mucronatus* Stål, 1859 (Santos et al., 2022). Given this, the first occurrence of *R. nasutus* in the city of Barra/BA increases the total number of species already found. Although there are no reports of *R. nasutus* occurrence in neighboring cities of the municipality, the presence of this species in the urban environment indicates its possible adaptation in this region, since it is typical of palms and carnaúbas, with reports of invasion and colonization in the intradomicile (Dias et al., 2008; Oliveira et al., 2009; Sarquis et al., 2012; Fidalgo et al., 2018; Barbosa-Silva et al., 2019).

In the natural environment, *R. nasutus* preferentially inhabits palms and bird and mammal nests but has demonstrated an increasing capacity for adaptation to artificial ecotopes, such as chicken coops, corrals, pigsties, and material stockpiles (Sarquis et al., 2012; Fidalgo et al., 2018; Barbosa-Silva et al., 2019), which implies a synanthropization process. The ecological plasticity of *R. nasutus* favors its approach to human dwellings, especially in areas where deforestation, burning, and agricultural expansion reduce the availability of sylvatic shelters (Freitas et al., 2017). In this study, the species was only found in the intradomicile, possibly due to the lack of hosts outside and the good physical structure of the residence. The active behavior of one of the observed specimens, flying in search of food, indicates a possible situation of opportunistic displacement motivated by the lack of food sources in the surroundings. These aspects reinforce the capacity of *R. nasutus* to invade urban environments and adapt to new ecological conditions (Parente et al., 2017; Cavalcante et al., 2020).

Studies in Rio Branco, Acre, demonstrate that urban residential complexes are frequently invaded by wild triatomine bugs, which can reach up to the 13th floor of buildings, indicating active dispersal by flight and attraction to artificial light sources (Ribeiro et al., 2019; Moura et al., 2024). In Manaus, Amazonas, intense flight activity of wild triatomine bugs in urban areas was also documented, with seasonal variation between dry and rainy periods, highlighting the role of climatic conditions in the dynamics of home invasion by these vectors (Naiff et al., 1998). Specifically for *R. nasutus*, studies have shown that human activities, such as deforestation, habitat fragmentation, and modification of wild ecotopes, favor closer contact with anthropogenic environments (Lima & Sarquis, 2008). The occurrence of *R. nasutus*, also reported here, fits into this broader context, corroborating the hypothesis of ecological plasticity and potential synanthropization, although without evidence of domestic colonization. Furthermore, recent records of other *Rhodnius* species in other urban areas of Brazil, such as the presence of *R. neglectus* in Araraquara, São Paulo, reinforce that urban invasion by triatomines is not restricted to specific regions or biomes, but represents a growing phenomenon of national relevance for entomological surveillance (Santana et al., 2024). In addition, these studies show that urban intradomiciliary records should not be interpreted merely as isolated accidental events and suggest that environmental and anthropogenic factors can promote the opportunistic dispersal of typically wild triatomines.

From an epidemiological point of view, *R. nasutus* presents behavioral characteristics that increase its potential to transmit *T. cruzi*, such as high host-seeking capacity, long feeding periods, and immediate defecation after the blood meal (Oliveira et al., 2009). Field studies point to natural infection rates between 11% and 25% in different localities (Sarquis et al., 2012; Lima et al., 2012), which is consistent with the detection of an infected specimen in this study. Thus, the urban record of *R. nasutus* in Barra stands out as a warning for strengthening entomological and environmental surveillance actions, especially in urban areas close to native palms and artificial ecotopes. Systematic inspection of these environments is also recommended, associated with health education and continuous monitoring of vector populations, to prevent domiciliary colonization and reduce the risk of vector transmission of Chagas disease (Gurgel-Gonçalves et al., 2012; Peretolchina et al., 2018). Therefore, new investigations must be conducted in the municipality to determine the specimen's occurrence sites, as well as to verify positivity for *T. cruzi*.

## Data Availability

The datasets generated and/or analysed during the current study are available from the corresponding author upon reasonable request.
